# The Use of Bayesian Networks to Assess the Quality of Evidence from Research Synthesis: 2. Inter-Rater Reliability and Comparison with Standard GRADE Assessment

**DOI:** 10.1371/journal.pone.0123511

**Published:** 2015-12-30

**Authors:** Alexis Llewellyn, Craig Whittington, Gavin Stewart, Julian PT Higgins, Nick Meader

**Affiliations:** 1 Centre for Reviews and Dissemination, University of York, York, United Kingdom; 2 Centre for Outcomes Research and Effectiveness Research, Department of Clinical, Educational and Health Psychology, University College London, London, United Kingdom; 3 School of Agriculture, Food and Rural Development, Newcastle University, Newcastle, United Kingdom; 4 School of Social and Community Medicine, University of Bristol, Bristol, United Kingdom; University of Illinois-Chicago, UNITED STATES

## Abstract

**Background:**

The grades of recommendation, assessment, development and evaluation (GRADE) approach is widely implemented in systematic reviews, health technology assessment and guideline development organisations throughout the world. We have previously reported on the development of the Semi-Automated Quality Assessment Tool (SAQAT), which enables a semi-automated validity assessment based on GRADE criteria. The main advantage to our approach is the potential to improve inter-rater agreement of GRADE assessments particularly when used by less experienced researchers, because such judgements can be complex and challenging to apply without training. This is the first study examining the inter-rater agreement of the SAQAT.

**Methods:**

We conducted two studies to compare: a) the inter-rater agreement of two researchers using the SAQAT independently on 28 meta-analyses and b) the inter-rater agreement between a researcher using the SAQAT (who had no experience of using GRADE) and an experienced member of the GRADE working group conducting a standard GRADE assessment on 15 meta-analyses.

**Results:**

There was substantial agreement between independent researchers using the Quality Assessment Tool for all domains (for example, overall GRADE rating: weighted kappa 0.79; 95% CI 0.65 to 0.93). Comparison between the SAQAT and a standard GRADE assessment suggested that inconsistency was parameterised too conservatively by the SAQAT. Therefore the tool was amended. Following amendment we found fair-to-moderate agreement between the standard GRADE assessment and the SAQAT (for example, overall GRADE rating: weighted kappa 0.35; 95% CI 0.09 to 0.87).

**Conclusions:**

Despite a need for further research, the SAQAT may aid consistent application of GRADE, particularly by less experienced researchers.

## Background

The Grades of Recommendation, Assessment, Development, and Evaluation (GRADE) is the most widespread method for rating the quality of evidence and grading recommendations in healthcare [1]. More than 65 international organisations have adopted the GRADE approach, and it is becoming an international standard. The GRADE approach for rating the quality of a body of evidence evaluates the evidence available for a given question and outcome with respect to several key domains. These include risk of bias, consistency of results, precision, directness of evidence, and publication bias. Evidence from randomised controlled trials is considered high quality (valid), but can be downgraded across domains. Observational studies begin at low quality evidence but their rating can be upgraded (provided no other limitations have been identified in the five domains) for three primary reasons: large magnitude of effect, evidence of a dose-response effect, confounders likely to minimize the effect. The process leads to a rating on the quality of evidence for each outcome of high, moderate, low or very low where high indicates that any future research is likely to be confirmatory.

A key advantage of GRADE is that it can lead to transparent judgments as a clear rationale is needed for each downgrade of the quality of evidence [1]. Another desirable feature of the approach is the ability to make complex nuanced judgments using a common framework that is not based solely on review process or the type of evidence included in the synthesis.

However, there are potential drawbacks to the use of GRADE. Application of GRADE can be complex, as implied by the lengthy series of guidance articles in the Journal of Clinical Epidemiology, currently standing at 15 papers [1]. This raises the possibility that multiple reviewers may differ in their decisions and justifications to upgrade/downgrade the quality of evidence. Although research conducted by the GRADE working group shows that two individual raters can reach substantial agreement when assessing the quality of evidence for a given outcome [2], others have found variable agreement in assessments within and across key domains among experienced reviewers [3].

The GRADE process may also be resource intensive. As use of GRADE is likely to increase (grading the evidence is now a recommended step within Cochrane systematic reviews [4]), tools which may improve the efficiency and replicability of the grading process in a resource-constrained decision-making environment are likely to be increasingly important.

To assist with GRADE assessments and address these potential issues, we have previously proposed a quality assessed tool based on a Bayesian network [5–6]. Bayesian networks, or directed acyclical graphs, are graphical statistical models comprising a network of nodes connected by directed links, with probability functions attached to each node to describe how other nodes linked to it influence its contents [7]. They have applications in quality control processes [8] and have been proposed as tools to assess internal validity in an evidence synthesis context and to facilitate a complex systems approach to evidence-synthesis using a variety of information sources and data types [8–9].

The SAQAT provides an assessment of overall quality of evidence by formalising the structure provided by the GRADE framework in a logic model and by giving a specific weight to each of the different items considered by GRADE. This paper has two aims. First, we assess inter-rater reliability of the SAQAT. Second, we compare the SAQAT with a standard GRADE assessment empirically, exploring reasons for disagreements between the two approaches, and identifying potential areas of improvement for the semi-automated tool.

## Methods

### Development of the Quality Assessment Tool

SAQAT comprises two parts: a checklist and a Bayesian network. We have described the development of these in detail in other articles [5–6]. Briefly, we developed a checklist of questions that underpin a GRADE assessment. Responses to these questions are then used to populate the Bayesian network. This semi-automated procedure produces probabilities of limitations within the five domains (risk of bias, imprecision, inconsistency, indirectness, publication bias). These are in the form of a probability distribution across potential judgements within the domain in the GRADE framework (e.g. for risk of bias, the probability that there were no limitations, serious limitations, or very serious limitations). These are then combined across domains to provide probabilistic statements about the overall quality of evidence contained in a systematic review (i.e. whether the quality evidence for the review was most likely high, moderate, low or very low).

Risk of bias was based on items in the Cochrane risk of bias tool [4]. Inconsistency was based primarily on visual assessment of forest plots and the statistical quantification of heterogeneity based on I^2^ and Q statistics. Indirectness included items on the applicability of the population, intervention, comparator, outcome, and whether conclusions were based on a direct comparison of groups. Imprecision was based on items on the width of the confidence interval in relation to minimally important difference and sample size. Publication bias included items on the comprehensiveness of the search strategy, whether included studies had industry influence, funnel plot asymmetry and whether there was evidence of discrepancies between published and unpublished trials (see Appendix 1 for a full list of items).

We have focused specifically on the context of randomised controlled trials (RCTs) where a meta-analysis has been conducted. Therefore we did not include factors to upgrade the evidence (magnitude of effect, dose-response relationship, adjusting for known confounding) as these are recommended for use only in the context of systematic reviews of non-randomised studies. Extension of the SAQAT for non-randomised studies is currently in progress.

### Study 1: Inter-rater reliability between two independent users of the SAQAT

Following the development and initial piloting of the checklist we conducted a more formal evaluation of its consistency. The inter-rater agreement for each of the checklist questions is reported in another study [6].

To evaluate the inter-rater agreement of the SAQAT, which combines the checklist with a Bayesian network, we examined a total of 29 meta-analyses from a purposive sample of systematic reviews of RCTs from the Database of Systematic Reviews of Effects (DARE). Papers were selected to ensure a diverse range of reviews from a variety of disease areas such as cardiology, diabetes, pulmonary disease, neurological conditions and oncology. Reviews on substance misuse, mental health and HIV prevention were also included. Both pharmacological and non-pharmacological interventions were considered. We also selected reviews with varying quality of reporting.

The checklist was used by two authors independently for all reviews. For each review a specific population, intervention, comparison and outcome (PICO) were selected beforehand to ensure both reviewers conducted the assessment on the same PICO elements of the review. GENIE software (freely downloadable from http://genie.sis.pitt.edu/) was used to calculate probability distributions in the Bayesian network.

We calculated weighted kappa statistics with their associated 95% confidence interval (CI) to compare agreement on ratings of quality of evidence. We interpreted the coefficients according to the following guidelines: below chance—poor; 0.01–0.20 slight agreement; 0.21–0.40 fair agreement; 0.41–0.60 moderate agreement; 0.61–0.80 substantial agreement; and 0.81–1 perfect agreement [10]. The following formula was used to calculate weights: *1- ((i-j) / (k-1)) ^2*, where i = rows, j = columns, and k = number of possible ratings.

### Study 2: Agreement between ratings of the SAQAT and a standard GRADE assessment

We next compared the judgements of our semi-automated tool with a standard GRADE assessment conducted by an experienced user of GRADE (CW).

#### Formal assessment

A purposive sample of 15 recent Cochrane reviews [11–25] in mental health was selected to reflect the area of expertise of the experienced GRADE user. A wide range of populations, interventions, comparators, and outcomes (PICO) were deliberately chosen to explore the application of the SAQAT in a variety of contexts. One set of PICO was selected for each review. All reviews had to present meta-analyses of RCTs, since the current tool is primarily designed for these types of reviews.

Quality of evidence was assessed for each review using the standard GRADE approach and the SAQAT by two reviewers independently. Assessments were made for the five main domains (risk of bias, consistency, directness, precision, publication bias), as well as overall quality of evidence. All received a rating of high, moderate, low, or very low quality.

#### GRADE

One reviewer (CW) conducted assessments for each review using standard GRADE criteria and recorded his judgements using GRADEPro software [26]. He is a long time member of the GRADE working group, author on papers summarising GRADE methods [27–28], and has over ten years’ experience in systematic review and guideline development.

#### SAQAT

One experienced systematic reviewer with no expert knowledge in mental health (AL) and no prior experience of conducting GRADE assessments assessed the quality of evidence using the standard list of questions from SAQAT. As in study 1, responses to the checklist questions were entered into the Bayesian network using GENIE software to calculate probability distributions for each domain and the overall quality for each review. We used these probability distributions to derive judgements in a format reflecting standard GRADE assessments.

#### Analysis

One author (NM) collated the judgements from SAQAT and the standard GRADE assessment and identified any differences in judgements. As in study 1, we calculated weighted kappas and 95% confidence intervals (CIs) between the standard GRADE and SAQAT assessments for each of the five key domains (risk of bias, consistency, precision, directness, and publication bias) and overall quality of evidence and used the same criteria and weights for evaluating the extent of agreement.

Where the assessors always or nearly always gave the same assessment for the domain, weighted kappas could not be calculated. Therefore we also presented the rate of agreements for each domain and the overall quality of evidence assessment. In addition, we presented a table comparing ratings from GRADE and the SAQAT and summarised these discrepancies narratively. We attempted to identify which disagreements were based on differences in raters’ judgments (e.g. what constitutes a clinically meaningful effect) from those based on properties inherent to the SAQAT (e.g. how much weight is given to “unclear” risk of bias).

Differences between standard GRADE and SAQAT assessments were discussed among three authors (NM, CW, AL) to understand the sources of the discrepancies.

#### Initial piloting of assessments and Bayesian network amendments

Before the formal evaluation we compared our responses on one systematic review. At this initial stage we identified a different interpretation of risk of bias between the Bayesian network and the judgement of the experienced GRADE user. Our Bayesian network, consistent with the Cochrane risk of bias tool [4], did not downgrade for risk of bias when there was insufficient information provided by authors. However, although the risk of bias domain of GRADE is largely based on the Cochrane tool, it was noted that the GRADE manual states that a reviewer may choose to downgrade when insufficient information is provided in the paper assessed. We decided to explore the impact of these differences in judgements about risk of bias in the formal stage of assessments as it reflects the reality of subtle differences in the interpretation and application of GRADE. It is also straightforward to parameterise the Bayesian network to reflect either assumption. The initial version of the Bayesian network did not downgrade the risk of bias domain due to unclear reporting. The network was then amended to reflect GRADE guidance more closely (downgrading for unclear reporting), and the analyses were re-run to explore the impact of this amendment on the results.

Our initial analysis also suggested the ratings of inconsistency generated by the Bayesian network were considered too conservative and lacked face validity. Therefore, the weight of the probabilities associated with two elements within the inconsistency domain (statistical heterogeneity and overlapping confidence intervals) and their interaction were amended. To reflect the effect of this change on the results for inconsistency and overall quality of evidence, weighted kappa scores were calculated before and after this amendment.

## Results

### Study 1: Comparison between SAQAT raters

#### Overall quality of evidence

For the overall judgement on quality of evidence there was substantial agreement between duplicate raters using the SAQAT. There was almost perfect agreement on risk of bias and imprecision factors; and substantial agreement for inconsistency, indirectness, and publication bias factors. Weighted kappa results are presented in Table 1.

**Table 1:** Comparison of raters’ judgements for two independent raters using the Semi-Automated Quality Assessment Tool (SAQAT).

Quality rating	Weighted Kappa (95% CI)	Magnitude of Agreement
Overall judgement	0.79 (0.65 to 0.93)	Substantial
Risk of Bias	1	Almost Perfect
Inconsistency	0.78 (0.62 to 0.95)	Substantial
Indirectness	0.69 (0.44 to 0.94)	Substantial
Imprecision	1	Almost Perfect
Publication bias	0.63 (0.17 to 1)	Substantial

#### Risk of bias

Although there was only moderate or low agreement between raters for some checklist items (selective reporting, no other bias, attrition bias) there was almost perfect agreement for the overall rating of risk of bias.

#### Imprecision

There was substantial agreement for all questions assessing imprecision and this was reflected in the almost perfect agreement for rating imprecision.

#### Indirectness

Agreement between raters on the applicability of populations and interventions was very low. However, there was substantial agreement on the use of surrogate outcomes and whether conclusions were based on direct comparisons. Despite the lack of agreement on questions of applicability there was almost perfect agreement on the overall ratings of indirectness. This reflects that the use of surrogate outcomes has most weight in generating the indirectness judgements.

#### Publication bias

There was substantial agreement for publication bias but this was lower than for all other domains. This might partly reflect less agreement on what constitutes a sufficient grey literature search and the difficulty in identifying publication biases.

### Study 2: Comparison between the use of SAQAT and a standard GRADE assessment

#### Quality rating judgments

Where calculable, magnitude of agreement between raters using SAQAT and standard GRADE assessment ranged from slight to moderate across domains before amending the Bayesian network within the SAQAT. After amending some parameters within the inconsistency domain of the tool and changing our assumptions about unclear reporting within the risk of bias domain, agreement across domains ranged from fair to moderate, and agreement on overall judgment increased from slight to fair (Table 2).

**Table 2:** Comparison of raters’ judgments for one rater using SAQAT and one rater using the standard GRADE approach (before and after SAQAT amendment).

Quality Rating domain	Weighted Kappa (95% CI)		Magnitude of Agreement	
	*Before amendment*	*After amendment*	*Before amendment*	*After amendment*
Risk of bias	0.13 (-0.22 to 0.48)	0.48 (0.01 to 0.94)	Slight	Moderate
Inconsistency	0.08 (-0.01 to 0.42)	0.30 (0 to 0.84)	Slight	Fair
Imprecision	0.47 (0.05 to 0.90)	0.47 (0.05 to 0.90)	Moderate	Moderate
Indirectness	Not estimable^1^	Not estimable^1^	N/A	N/A
Publication bias	Not estimable^2^	Not estimable^2^	N/A	N/A
**Overall judgment**	**0.11 (-0.34 to 0.47)**	**0.35 (-0.09 to 0.87)**	**Slight**	**Fair**

^1^ One discrepancy out of 15 judgments (93% agreement)

^2^ No discrepancies but only one review was rated as publication bias suspected (100% agreement)

After amending the tool, agreement on the risk of bias domain increased from slight to moderate, agreement on the inconsistency domain increased from slight to fair,. Rating of other domains remained unchanged (Table 2). The precision of the estimates was low due to the relatively limited number of reviews that were assessed, as shown by the wide confidence intervals.

#### Sources of disagreements between GRADE and SAQAT ratings

A full report of all rating decisions and brief commentaries on all disagreement between GRADE and SAQAT raters before and after Bayesian network amendment is presented in Appendix 2. These disagreements are summarised below.

#### Overall quality of evidence

Before amending the tool, twelve disagreements were recorded. The ratings generated by the Bayesian network were more conservative in nine of those, and in three cases, judgments differed by more than one level (moderate versus very low). After amending the inconsistency domain within the Bayesian network, the number of disagreements was reduced to seven, and one differed by more than one level. In nearly all disagreements, the overall quality of evidence ratings generated by SAQAT were more conservative than those made by the manual GRADE assessor.

In several cases, differences in severity between ratings appeared to be due to an accumulation of probability for downgrading across domains within the Bayesian network. With the manual GRADE assessor, quality of evidence ratings were generally based on the sum of downgrading scores from all domains (i.e. zero, minus one or minus two based on the perceived severity of limitations within each domain). Limitations that were considered too small to justify downgrading of a domain were less likely to be taken into account in the overall assessment. On the other hand, overall quality of evidence rating generated by the Bayesian network were affected by the accumulation of all identified limitations within each domain, however small.

#### Risk of bias

Disagreements on rating of risk of bias took place in nine out of 15 assessments. On three occasions, the GRADE rater downgraded the evidence due to unclear risk of bias, whereas SAQAT did not. As discussed above, this reflected a priori differences in interpretation of GRADE. The tool was amended to reflect GRADE guidance on downgrading for unclear reporting. After this, the number of disagreements was reduced to five.

Other disagreements reflected differences in judgment between raters about whether risk of bias was present and/or sufficiently prevalent to justify downgrading. One of the GRADE rater’s decisions to downgrade was a context-specific judgment based on knowledge of treatment area. Where disagreements occurred, the GRADE rater’s judgments were generally more conservative than the SAQAT rater’s. All disagreements differed by one level.

#### Inconsistency

Before amending the tool, disagreements about inconsistency were recorded for eight assessments. SAQAT was always more conservative, and in five cases, judgments differed by three levels (i.e. no serious inconsistency to very serious inconsistency). The weight of the probabilities associated with statistical heterogeneity, overlapping confidence intervals and their interaction were amended. Following this, the number of disagreements was reduced to five, and none differed by more than one level. The most common disagreement was based on whether variability of study estimates and effect direction were clinically meaningful and therefore a sufficient reason to downgrade the quality of evidence.

#### Imprecision

Only three disagreements on imprecision were recorded. In two cases, the disagreements suggested that the Bayesian network departed from the GRADE manual. The third disagreement was based on differing interpretations of what constituted a clinically meaningful effect. All disagreements differed by one level.

#### Indirectness

Only one disagreement on indirectness was recorded. The disagreement was probably due to differences in knowledge of subject area between the raters (difference of one level).

#### Publication bias

There was no disagreement between raters in this domain. This is likely to be due to the lack of evidence of publication bias for any of the outcomes assessed.

## Discussion

### Summary of main findings

When comparing two independent reviewers using SAQAT we found substantial agreement for overall quality and substantial to almost perfect agreement for all of the five domains of GRADE (risk of bias, imprecision, inconsistency, indirectness, publication bias). Agreement between GRADE and SAQAT on overall quality of evidence ratings was slight, and became fair after amending assumptions about risk of bias and inconsistency within the Bayesian network. Ratings generated by the Bayesian network were sometimes more conservative than manual GRADE assessments.

### Strengths and limitations

This is the first empirical and systematic study comparing the standard GRADE approach with a semi-automated approach incorporating the elements of GRADE into a Bayesian network. This study adds to the evidence that the SAQAT could be useful in the application of the GRADE approach.

A key advantage of SAQAT is its replicability. Our study shows that SAQAT can be applied transparently and consistently by two reviewers assessing the quality of evidence from a range of systematic reviews of RCTs. Therefore, the Quality Assessment Tool may be a promising tool to improve potential replicability issues observed in previous empirical comparisons between manual GRADE assessors, where agreement between raters was sometimes more variable [3].

Results on the comparison between SAQAT and a standard GRADE assessment are more mixed. Agreement between the two approaches varied across domains, although amendments made to assumptions within the Bayesian network improved levels of agreement. The precision of the weighted kappa estimates was low, primarily due to the relatively small sample size. Also, it is unclear how SAQAT compares to standard GRADE assessment in other health fields. Therefore further testing of the reliability of the tool using larger samples and in other areas is needed to confirm our findings.

Where disagreements about overall quality of evidence occurred, ratings generated by SAQAT tended to be more conservative. This may be because, unlike manual GRADE assessment, the Bayesian network automatically takes into account the accumulation of multiple limitations in the evidence, however small. Therefore this may reflect the strength of SAQAT approach when evaluating systematic review evidence where there are multiple small probabilities of limitations in the evidence.

**Fig 1:**
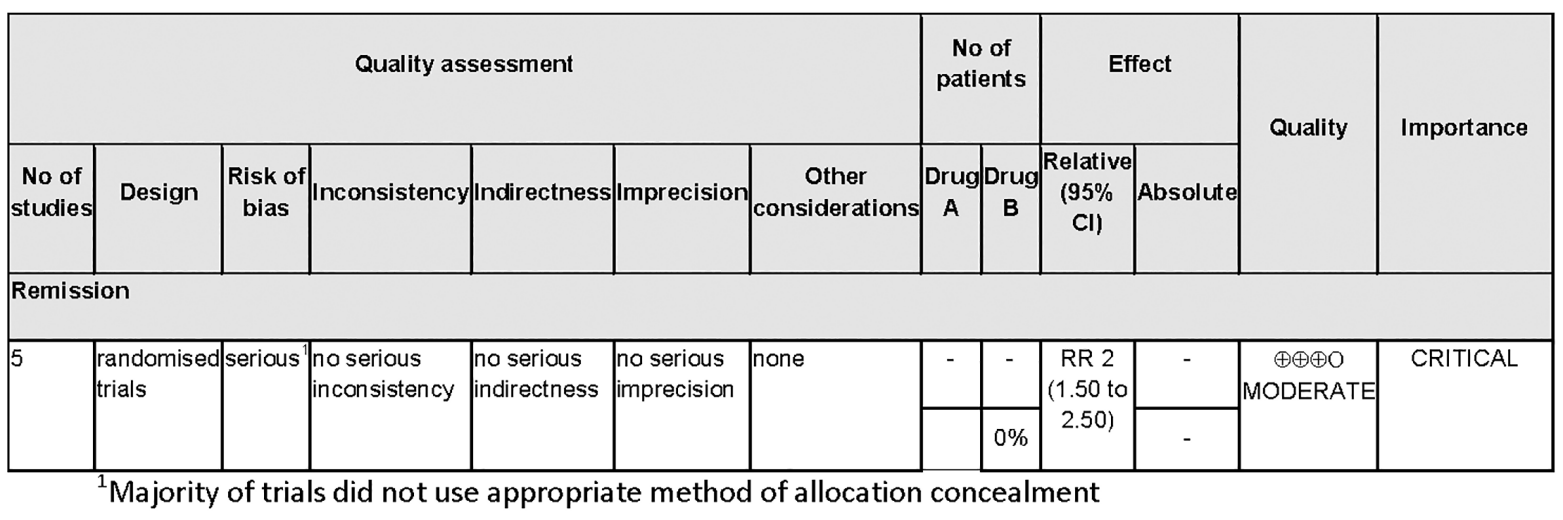
Example evidence profile using GRADEpro.

**Fig 2:**
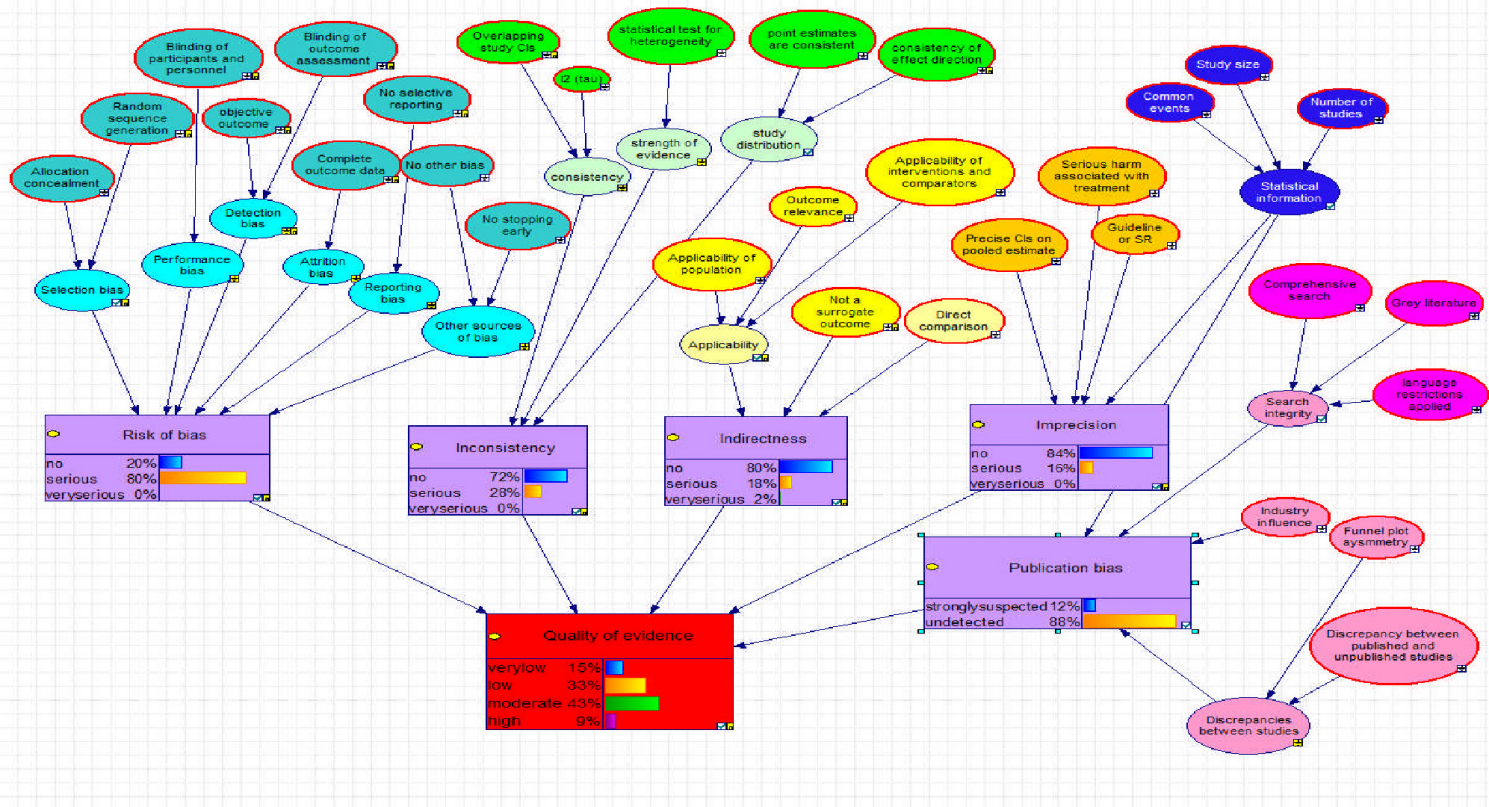
Example evidence profile with probability distributions from Bayesian network.

For the analyses, only the highest probability provided by the Bayesian network was taken into account. Evidence profiles can also be presented as probability distributions representing the uncertainty underlying these judgements. These figures may be presented alongside or as an alternative to standard evidence profiles produced by GRADE Pro (see Fig 1 and Fig 2).

### Suggestions for further research

The current version of SAQAT is applicable to systematic reviews that include meta-analyses of RCTs. The GRADE approach can also be applied to meta-analyses of observational studies and narrative approaches to synthesising evidence. Further work is currently in progress to adapt and validate our approach for such reviews.

Despite numerous publications about the GRADE approach and widespread use, there remain few empirical and systematic evaluations of its application. Further research on how SAQAT compares to standard GRADE assessments in other medical fields and with bigger samples is required to confirm the findings of this study.

Some aspects of the GRADE framework are better supported by evidence than others. Further changes to the structure and parameterisation of SAQAT would allow qualitative and quantitative exploration of the potential weight and impact of (and interaction between) specific elements within domains, including those elements that are less certain. Methodological applications could involve mapping probabilities of bias and applying them, or formally eliciting expert opinion to generate distributions for the probabilities reflecting uncertainty appropriately.

### Suggestions for further practice

Use of SAQAT is probably most likely to benefit those with limited experience of using GRADE. However, given the complexity of GRADE, we think that experienced users of this approach may also benefit from a reminder of key criteria to help ensure consistent and repeatable quality of evidence ratings. We also think that institutions considering adopting GRADE, but concerned about the resource and training implications, may also benefit.

A further benefit of using SAQAT is the potential in the future to automate the entire process of validity assessment. For example, nodes can be parameterised using text mining software or data stored by document management systems used for review production and storage (such as ARCHIE used by the Cochrane Collaboration). The benefits of automation are gaining more attention as a response to the challenges of conducting increasingly large and complex evidence syntheses in a timely manner to inform decision makers.

## Conclusions

The Semi-Automated Quality Assessment Tool (SAQAT), a checklist of questions and a Bayesian network designed to model judgements according to GRADE criteria, can produce consistent and reproducible judgements about the quality of evidence from meta-analyses of RCTs. Despite a need for further research, SAQAT may aid consistent application of GRADE, particularly by less experienced reviewers.

## Supporting Information

S1 Table Quality Assessment Tool checklist items. (DOCX)

S2 Table Overview of agreements between SAQAT and standard GRADE ratings. (DOCX)
