# Supplementary material for: The Use of Bayesian Networks to Assess the Quality of Evidence from Research Synthesis: 2. Inter-Rater Reliability and Comparison with Standard GRADE Assessment
Source: PLoS One. 2015 Dec 30;10(12):e0123511. doi: 10.1371/journal.pone.0123511 (PMC4696848; doi:10.1371/journal.pone.0123511)
Supplement: S1 Table — (DOCX) [file pone.0123511.s001.docx]

**S1 Table. Quality Assessment Tool checklist items**

| **RISK OF BIAS (1)** | **RISK OF BIAS (2)** | **INCONSISTENCY** | **INDIRECTNESS** | **IMPRECISION** | **PUBLICATION BIAS** |
| --- | --- | --- | --- | --- | --- |
| Was random sequence generation used (i.e. no potential for selection bias)? | Were more than (80%)^*^ of participants enrolled in trials included in the analysis (i.e. no potential for attrition bias)? | Point estimates do not vary widely (i.e. no clinical meaningful inconsistency)? | Are the populations in included studies applicable to the target population? | Is the confidence interval for the pooled estimate not consistent with benefit and harm? | Did the authors conduct a comprehensive search? |
| Was Allocation concealment used (i.e. no potential for selection bias)? | Was data reported consistently for the outcome of interest (selective reporting)? | To what extent do confidence intervals overlap? | Are the interventions in included studies applicable to target intervention? | What is the magnitude of the median sample size? | Did the authors search for grey literature? |
| Was there blinding of participants and personnel (i.e. no potential for performance bias)? | No other biases reported (i.e. no potential of other bias)? | Is the direction of effect consistent? | Is the included outcome not a surrogate outcome? | What is the magnitude of the number of included studies? | Did the authors apply no restrictions on the basis of language? |
| Was there blinding of outcome assessment (i.e. no potential for detection bias)? | Did the trial end as scheduled (i.e. was not stopped early)? | What is the magnitude of statistical heterogeneity (as measured by I^2^)? | Is the outcome timeframe sufficient? | Is the outcome a common event?  (i.e. occurs more than 1/100)* | There was no industry influence on studies included in the review? |
| Was an objective outcome used? |  | Was the test for heterogeneity statistically significant (p<0.1)? | Are the conclusions of the study based on direct comparisons? | Is there no evidence of serious harm associated with treatment? | There was no evidence of funnel plot asymmetry? |
|  |  |  |  |  | Was there no discrepancy in findings between published and unpublished trials? |
