# Supplementary material for: The Use of Bayesian Networks to Assess the Quality of Evidence from Research Synthesis: 2. Inter-Rater Reliability and Comparison with Standard GRADE Assessment
Source: PLoS One. 2015 Dec 30;10(12):e0123511. doi: 10.1371/journal.pone.0123511 (PMC4696848; doi:10.1371/journal.pone.0123511)
Supplement: S2 Table — (DOCX) [file pone.0123511.s002.docx]

**S2 Table. Overview of agreements between Quality Assessment Tool (QAT) and standard GRADE ratings**

| **Study id** | **Risk of bias (before changes)** | **Risk of bias (after changes)** | **Inconsistency (before changes)** | **Inconsistency (after changes)** | **Imprecision** | **Indirectness** | **Publication bias** | **Overall (before changes)** | **Overall (after changes)** | **Comments** |
| --- | --- | --- | --- | --- | --- | --- | --- | --- | --- | --- |
| 1 Archer | No | No | Manual: no  SAQAT: very serious | Manual: no  SAQAT: serious | No | Manual: serious  SAQAT: no | No | Manual: Moderate  SAQAT: low | Moderate | Indirectness: Manual rater probably using knowledge of field judged that there was serious indirectness (many studies either included or didn't explicitly exclude patients with a chronic physical health condition).  Inconsistency: SAQAT rater downgraded based on variability of study estimates and effect direction - difference in judgement whether variability was clinically meaningful |
| 2 Cipriani | No | No | Manual: serious  SAQAT: very serious | Serious | No | No | No | Manual: Moderate  SAQAT: very low | Manual: Moderate  SAQAT: low | Overall: SAQAT accumulation of probability for downgrading across domains |
| 3 Baumeister | Manual: serious (selective reporting)  SAQAT: no | Manual: serious (selective reporting)  SAQAT: no | No | No | Manual: serious  SAQAT: no | No | No | Manual: low  SAQAT: moderate | Manual: low  SAQAT: moderate | Risk of bias: manual GRADE context specific judgment based on knowledge of treatment area regarding potential selective reporting  Imprecision: manual rater used more conservative criterion than SAQAT (more consistent with GRADE manual concerning a limited number of events in the analysis) |
| 4 Williams | Manual: serious  SAQAT: very serious | Manual: serious  SAQAT: very serious | Manual: no  SAQAT: very serious | Manual: no  SAQAT: serious | Manual: no  SAQAT: serious | No | No | Manual: Moderate  SAQAT: very low | Manual: moderate  SAQAT: very low | Risk of bias: difference in judgement  Inconsistency: SAQAT downgrade for varying study estimates and inconsistent effect direction  Imprecision: difference in judgement concerning whether lower CI estimate constitutes a clinical meaningful difference |
| 5 Depping | Manual: serious  SAQAT: no | Manual: serious  SAQAT: serious | Serious | Serious | No | No | No | Manual: low  SAQAT: moderate | Low | Risk of Bias: unclear only downgraded in modified version of SAQAT |
| 6 Soomro | Manual: serious  SAQAT: no | Serious | Manual: no  SAQAT: serious | No | No | No | No | Manual: Moderate  SAQAT: low | Moderate | Risk of Bias: manual rater downgraded for risk of bias where there was some evidence of bias identified in addition to unclear reporting  SAQAT rater also reaches a moderate rating in the amended version following change of assumptions about risk of bias and inconsistency. |
| 7 Rolinski | Serious | Serious | No | No | No | No | No | Low | Low | Manual rater initially had an overall judgement of moderate. However after discussion agreed there was sufficient basis for further downgrade due to multiple problems across domains. |
| 8 Matar | Manual: serious  SAQAT: no | Manual: serious  SAQAT: no | No | No | Serious | No | No | Low | Low | Risk of Bias: manual rater downgraded for risk of bias where there was some evidence of bias identified in addition to unclear reporting - difference in judgement between raters |
| 9 James | No | No | No | No | Manual: Serious  SAQAT: No | No | No | Moderate | Moderate | Imprecision: manual rater used more conservative criterion than SAQAT (more consistent with GRADE manual i.e. there were a limited number of events)  SAQAT also resulted in a rating of moderate due to accumulation of probabilities of downgrading across domains |
| 10 Gillies | Manual: serious  SAQAT: no | Manual: serious  SAQAT: no | Manual: no  SAQAT: serious | No | Serious | No | No | Manual: low  SAQAT: very low | Low | Risk of Bias: manual rater downgraded for risk of bias where there was some evidence of bias identified in addition to unclear reporting - difference in judgement between raters  SAQAT also resulted in an overall rating of moderate due to accumulation of probabilities of downgrading across domains |
| 11 Pani | No | No | Manual: no  SAQAT: very serious | Manual: no  SAQAT: serious | Serious | No | Manual: serious  SAQAT: serious | Manual: low  SAQAT: very low | Manual: low  SAQAT: very low | Inconsistency: SAQAT downgrade for inconsistent effect direction and varying study estimates -disagreement between raters whether the variability was of a clinically meaningful magnitude |
| 12 Dietrich | Manual: no  SAQAT: serious | Manual: no  SAQAT: serious | No | No | No | No | No | Manual: high  SAQAT: moderate | Manual: high  SAQAT: moderate | Risk of bias: SAQAT downgraded due to potential performance bias and selective reporting |
| 13 Kisely | Manual: serious  SAQAT: no | Manual: serious  SAQAT: serious | No | No | Serious | No | No | Manual: Low  SAQAT:Moderate | Low | Risk of Bias: unclear downgraded only in modified SAQAT. |
| 14 Vernooij-Dassen | Serious | Serious | Manual: no  SAQAT: very serious | Manual: no  SAQAT: serious | Serious | No | No | Manual: low  SAQAT: very low | Manual: low  SAQAT: very low | Inconsistency: SAQAT rater downgraded based on variability of study estimates and effect direction - difference in judgement whether variability was clinically meaningful |
| 15 Clearly | Manual: serious  SAQAT: no | Manual: serious  SAQAT: serious | Manual: no  SAQAT: very serious | Manual: no  SAQAT: serious | Serious | No | No | Manual: moderate  SAQAT: very low | Manual: low  SAQAT: very low | Risk of bias: unclear only downgraded in modified SAQAT.  Inconsistency:  SAQAT rater downgraded based on variability of study estimates and effect direction - difference in judgement whether variability was clinically meaningful |
| Total disagreements | 9/15  (7/15 manual rater more conservative, 2/15 SAQAT more conservative)  Of those, 3/15 disagreements based manual rater but not SAQAT downgrading for poor reporting | 5/15  (3/15 manual rater more conservative, 2/15 SAQAT more conservative) | 8/15  (SAQAT always more conservative  - 5 by two levels (e.g. no inconsistency vs very serious inconsistency)  -3 by one level) | 5/15  (SAQAT always more conservative) | 3/15  (2/15 manual rater more conservative, 1/15 SAQAT rater) | 1/15  (Probably due to differences in knowledge of subject area by manual rater) | 0/15 | 12/15  (9/15 SAQAT more conservative) | 7/15  (6/15 SAQAT more conservative, 1/15 manual rating more conservative) |  |
